# Supplementary material for: Large-scale examination of early-age sex differences in neurotypical toddlers and those with autism spectrum disorder or other developmental conditions
Source: Nat Hum Behav. 2025 May 26;9(8):1697–709. doi: 10.1038/s41562-025-02132-6 (PMC12367553; doi:10.1038/s41562-025-02132-6)
Supplement: Supplementary file 1 — Supplementary Tables 1–18 and Fig. 1. [file 41562_2025_2132_MOESM1_ESM.pdf]

nature human behaviour

Supplementary information https://doi.org/10.1038/s41562-025-02132-6

Large-scale examination of early-age sex differences in neurotypical toddlers and those with autism spectrum disorder or other developmental conditions

In the format provided by the authors and unedited

Supplementary Information

Supplementary Table 1. Two-way ANOVA of sex and group across test subscales.

Test	*Subscale*	*Effect*	*F-ratio*	*DFn*	*DFd*	*GES*	*P-value*
ADOS	Social Affect	Sex	0.02	1	1801	0.00	0.891
		Group	2717.32	1	1801	0.60	< .001*
		Sex × Group	5.41	1	1801	0.00	0.02*
	Restricted & Repetitive Behavior	Sex	1.84	1	1801	0.00	0.175
		Group	1935.8	1	1801	0.52	< .001*
		Sex × Group	0.07	1	1801	0.00	0.790
	Overall Total	Sex	0.14	1	1801	0.00	0.712
		Group	3488.96	1	1801	0.66	< .001*
		Sex × Group	3.32	1	1801	0.00	0.069
Vineland	Communication	Sex	4.46	1	1803	0.00	0.035*
		Group	1240.66	1	1803	0.41	< .001*
		Sex × Group	0.22	1	1803	0.00	0.638
	Daily Living Skills	Sex	18.04	1	1803	0.01	< .001*
		Group	642.34	1	1803	0.26	< .001*
		Sex × Group	1.57	1	1803	0.00	0.211
	Motor Skills	Sex	0	1	1803	0.00	0.991
		Group	251.29	1	1803	0.12	< .001*
		Sex × Group	0.04	1	1803	0.00	0.842
	Socialization	Sex	6.14	1	1803	0.00	0.013*
		Group	1106.3	1	1803	0.38	< .001*
		Sex × Group	0.39	1	1803	0.00	0.530
	Adaptive Behavior Composite	Sex	8.27	1	1803	0.01	0.004*
		Group	1327.82	1	1803	0.42	< .001*
		Sex × Group	0.76	1	1803	0.00	0.383
MSEL	Fine Motor	Sex	4.37	1	1441	0.00	0.037*
		Group	409.25	1	1441	0.22	< .001*
		Sex × Group	0.17	1	1441	0.00	0.683
	Visual Reception	Sex	0.54	1	1441	0.00	0.462
		Group	793.86	1	1441	0.36	< .001*
		Sex × Group	5.65	1	1441	0.00	0.018*
	Receptive Language	Sex	0.56	1	1441	0.00	0.456
		Group	1185.1	1	1441	0.45	< .001*
		Sex × Group	5.85	1	1441	0.00	0.016*
	Expressive Language	Sex	2.81	1	1441	0.00	0.094
		Group	1261.54	1	1441	0.47	< .001*
		Sex × Group	0.05	1	1441	0.00	0.821
CDI-WG	Words Produced	Sex	3.69	1	461	0.01	0.055

		Group	34.58	1	461	0.07	< .001*
		Sex × Group	0.71	1	461	0.00	0.401
	Words Understood	Sex	5.23	1	461	0.01	0.023*
		Group	53.68	1	461	0.10	< .001*
		Sex × Group	0.26	1	461	0.00	0.609
	Early Gestures	Sex	3.1	1	461	0.01	0.079
		Group	99.4	1	461	0.18	< .001*
		Sex × Group	0.36	1	461	0.00	0.548
	Later Gestures	Sex	5.6	1	461	0.01	0.018*
		Group	80.94	1	461	0.15	< .001*
		Sex × Group	2.22	1	461	0.01	0.137
	Total Gestures	Sex	5.49	1	461	0.01	0.020*
		Group	100.31	1	461	0.18	< .001*
		Sex × Group	1.7	1	461	0.00	0.193
CDI-WS	Words Produced	Sex	7.46	1	888	0.01	0.006*
		Group	430.87	1	888	0.33	< .001*
		Sex × Group	9.11	1	888	0.01	0.003*
CSBS	Social	Sex	11.31	1	910	0.01	0.001*
		Group	62.2	1	910	0.06	< .001*
		Sex × Group	0.02	1	910	0.00	0.883
	Speech	Sex	7.43	1	910	0.01	0.007*
		Group	65.86	1	910	0.07	< .001*
		Sex × Group	0.41	1	910	0.00	0.521
	Symbolic	Sex	10.59	1	910	0.01	0.001*
		Group	65.44	1	910	0.07	< .001*
		Sex × Group	0.07	1	910	0.00	0.791
	Total	Sex	11.81	1	910	0.01	0.001*
		Group	74.36	1	910	0.08	< .001*
		Sex × Group	0.02	1	910	0.00	0.902
Geopref	Social % Fixation	Sex	0.28	1	964	0.00	0.594
		Group	121.27	1	964	0.112	< .001*
		Sex × Group	0.06	1	964	0.00	0.808

Note. * = *P* < .05, *DFn* = Degrees of freedom for numerator, *DFd* = Degrees of freedom for denominator, *GES* = Generalized Eta Squared (effect size). All the tests are two-sided.

Supplementary Table 2. Methods to determine optimal number of clusters.

Index	*Index value*	*Best # of Clusters*	*Index*	*Index value*	*Best # of Clusters*
KL	16.54	6	Duda	1.18	2
CH	1441.47	2	PseudoT2	-132.21	2
Hartigan	173.94	3	Beale	-0.69	2
CCC	-1.26	2	Ratkowsky	0.50	2
Scott	997.74	3	Ball	1041.42	3
Marriot	3.02E+18	3	PtBiserial	0.63	2
TrCovW	162194.57	3	Frey	1.87	3
TraceW	512.06	3	McClain	0.55	2
Friedman	3.16	3	Dunn	0.06	8
Rubin	-0.22	3	SDindex	1.61	2
Cindex	0.25	5	SDbw	0.26	10
DB	0.96	2	BIC	-18132.68	3
Silhouette	0.42	2	Elbow method	Graphical	3

Supplementary Table 3. Cluster separation in train set.

Domain	*Subscale*	*Effect*	*F-ratio*	*DFn*	*DFd*	*GES*	*P-value*
Social	ADOS-Social Affect	Clusters	1239.81	2	1334	0.65	< .001*
	Vineland-Socialization	Clusters	996.58	2	1334	0.60	< .001*
Motor	Vineland-Motor Skills	Clusters	317.66	2	1334	0.32	< .001*
	MSEL-Fine Motor	Clusters	713.9	2	1334	0.52	< .001*
Language	Vineland-Communication	Clusters	1377.6	2	1334	0.67	< .001*
	MSEL-Receptive Language	Clusters	1159.82	2	1334	0.64	< .001*
	MSEL-expressive language	Clusters	1003.56	2	1334	0.60	< .001*

Note. * = $P < .05$, *DFn* = Degrees of freedom for numerator, *DFd* = Degrees of freedom for denominator, *GES* = Generalized Eta Squared (effect size). All the tests are two-sided.

Supplementary Table 4. Cluster pairwise comparisons in train set.

Domain	*Subscale*	*Cluster*		
			1	2
			P-value	P-value
Social	ADOS-Social Affect	2	< .001*	NA
		3	< .001*	< .001*
	Vineland-Socialization	2	< .001*	NA
		3	< .001*	< .001*
Motor	Vineland-Motor Skills	2	< .001*	NA
		3	< .001*	< .001*
	MSEL-Fine Motor	2	< .001*	NA
		3	< .001*	< .001*
Language	Vineland-Communication	2	< .001*	NA
		3	< .001*	< .001*
	MSEL-Receptive Language	2	< .001*	NA
		3	< .001*	< .001*
	MSEL-expressive language	2	< .001*	NA
		3	< .001*	< .001*

Note. Statistic is Kruskal-Wallis chi-squared test and all the tests are two-sided. Multiple pairwise comparisons were corrected by FDR, * = $P < .05$.

Supplementary Table 5. Cluster separation in test set.

Domain	*Subscale*	*Effect*	*F-ratio*	*DFn*	*DFd*	*GES*	*P-value*
Social	ADOS-Social Affect	Clusters	316.36	2	333	0.66	< .001*
	Vineland-Socialization	Clusters	216.56	2	333	0.57	< .001*
Motor	Vineland-Motor Skills	Clusters	66.36	2	333	0.29	< .001*
	MSEL-Fine Motor	Clusters	126.84	2	333	0.43	< .001*
Language	Vineland-Communication	Clusters	301.14	2	333	0.64	< .001*
	MSEL-Receptive Language	Clusters	200.71	2	333	0.55	< .001*
	MSEL-expressive language	Clusters	189.04	2	333	0.53	< .001*

Note. * = $P < .05$, *DFn* = Degrees of freedom for numerator, *DFd* = Degrees of freedom for denominator, *GES* = Generalized Eta Squared (effect size). All the tests are two-sided.

Supplementary Table 6. Cluster pairwise comparisons in test set.

Domain	*Subscale*	*Cluster*		
			1	2
			P-value	P-value
Social	ADOS-Social Affect	2	< .001*	NA
		3	< .001*	< .001*
	Vineland-Socialization	2	< .001*	NA
		3	< .001*	< .001*
Motor	Vineland-Motor Skills	2	< .001*	NA
		3	< .001*	< .001*
	MSEL-Fine Motor	2	< .001*	NA
		3	< .001*	< .001*
Language	Vineland-Communication	2	< .001*	NA
		3	< .001*	< .001*
	MSEL-Receptive Language	2	< .001*	NA
		3	< .001*	< .001*
	MSEL-expressive language	2	< .001*	NA
		3	< .001*	< .001*

Note. Statistic is Kruskal-Wallis chi-squared test and all the tests are two-sided. Multiple pairwise comparisons were corrected by FDR, * = $P < .05$.

Supplementary Table 7. Silhouette score.

	Cluster	*Sample size*	*Average silhouette width*
Train data	1	515	0.69
	2	680	0.23
	3	142	0.75
Test data	1	122	0.69
	2	203	0.21
	3	11	0.88

Supplementary Table 8. Cluster separation across external variables.

External Variable	*Effect*	*F-ratio*	*DFn*	*DFd*	*GES*	*P-value*
CDI-WG-Words Produced	Clusters	9.05	2	369	0.05	< .001*
CDI-WG-Words Understood	Clusters	12.28	2	369	0.06	< .001*
CDI-WG-Early Gestures	Clusters	35.15	2	369	0.16	< .001*
CDI-WG-Later Gestures	Clusters	17.85	2	369	0.09	< .001*
CDI-WG-Total Gestures	Clusters	26.38	2	369	0.13	< .001*
CDI-WS-Words Produced	Clusters	154.32	2	609	0.34	< .001*
Geopref-Social % Fixation	Clusters	117.06	2	738	0.24	< .001*
ADOS-Restricted and Repetitive Behavior	Clusters	703.05	2	1334	0.51	< .001*

Note. * = $P < .05$, *DFn* = Degrees of freedom for numerator, *DFd* = Degrees of freedom for denominator, *GES* = Generalized Eta Squared (effect size). All the tests are two-sided.

Supplementary Table 9. Cluster pairwise comparisons in external variables.

Eternal Variable	*Cluster*	1	2
		P-value	P-value
CDI-WG-Words Produced	2	< .001*	NA
	3	0.017*	0.379
CDI-WG-Words Understood	2	< .001*	NA
	3	0.037*	0.407
CDI-WG-Early Gestures	2	< .001*	NA
	3	0.003*	0.301
CDI-WG-Later Gestures	2	< .001*	NA
	3	0.004*	0.185
CDI-WG-Total Gestures	2	< .001*	NA
	3	0.001*	0.132
CDI-WS-Words Produced	2	< .001*	NA
	3	< .001*	< .001*
Geopref-Social % Fixation	2	< .001*	NA
	3	< .001*	< .001*
ADOS-Restricted and Repetitive Behavior	2	< .001*	NA
	3	< .001*	< .001*

Note. All the tests are two-sided and statistic is either Kruskal-Wallis chi-squared test or t-test where appropriate. Multiple pairwise comparisons were corrected by FDR, * = $P < .05$.

Supplementary Table 10 - Sex differences in SNF- train set.

Group	*Subscale*	*Cluster*	*Statistic*[a]	*P-value*	*Effect size*
ASD	ADOS-Sociafl Affect	1	1.75	0.185	-
	Vineland-Socialization	1	1.86	0.173	-
	Vineland-Motor Skills	1	-0.83[b]	0.411	-
	MSEL-Fine Motor	1	1.16[b]	0.254	-
	Vineland-Communication	1	0.26	0.610	-
	MSEL-Receptive Language	1	-1.26[b]	0.217	-
	MSEL-expressive language		0.23	0.635	-
TD	ADOS-Social Affect	1	0.41	0.522	-
	Vineland-Socialization	1	8.89	0.003*	0.02
	Vineland-Motor Skills	1	5.00	0.025*	0.01
	MSEL-Fine Motor	1	18.48	< .001*	0.04
	Vineland-Communication	1	2.71	0.100	-
	MSEL-Receptive Language	1	3.98[b]	< .001	0.4
	MSEL-expressive language	1	1.90	0.168	-
ASD	ADOS-Social Affect	2	7.23	0.007*	0.01
	Vineland-Socialization	2	7.95	0.005*	0.01
	Vineland-Motor Skills	2	0.37	0.544	-
	MSEL-Fine Motor	2	2.38	0.123	-
	Vineland-Communication	2	3.27	0.071	-
	MSEL-Receptive Language	2	2.36	0.124	-
	MSEL-expressive language	2	0.02	0.877	-
TD	ADOS-Social Affect	2	-1.75[b]	0.099	-
	Vineland-Socialization	2	0.98[b]	0.343	-
	Vineland-Motor Skills	2	-0.45[b]	0.658	-
	MSEL-Fine Motor	2	-0.36[b]	0.723	-
	Vineland-Communication	2	3.31	0.069	-
	MSEL-Receptive Language	2	-0.2[b]	0.841	-
	MSEL-expressive language	2	4.15	0.042*	0.20
ASD	ADOS-Social Affect	3	0.08	0.781	-
	Vineland-Socialization	3	1.79	0.182	-
	Vineland-Motor Skills	3	-0.69[b]	0.493	-
	MSEL-Fine Motor	3	1.09[b]	0.277	-
	Vineland-Communication	3	3.32	0.068	-
	MSEL-Receptive Language	3	0.34	0.558	-
	MSEL-expressive language	3	3.02	0.082	-

Note. [a] = Statistic is Kruskal-Wallis chi-squared test and its reported effect size is Eta squared. [b] = Statistic is t-test and its reported effect size is Cohen's *d*, * = $P < .05$. All the tests are two-sided.

Supplementary Table 11 – Sex differences in SNF- test set.

Group	*Subscale*	*Cluster*	*Statistic[a]**	*P-value*	*Effect size*
ASD	ADOS-Social Affect	1	0.72[b]	0.540	-
	Vineland-Socialization	1	-0.06[b]	0.954	-
	Vineland-Motor Skills	1	0.51[b]	0.641	-
	MSEL-Fine Motor	1	1.56[b]	0.227	-
	Vineland-Communication	1	4.54[b]	< .001*	1.25
	MSEL-Receptive Language	1	1.65[b]	0.228	-
	MSEL-expressive language		2.16[b]	0.045*	0.62
TD	ADOS-Social Affect	1	1.39	0.239	-
	Vineland-Socialization	1	1.11	0.292	-
	Vineland-Motor Skills	1	0.07	0.798	-
	MSEL-Fine Motor	1	0.80	0.370	-
	Vineland-Communication	1	0.26	0.613	-
	MSEL-Receptive Language	1	0.16	0.686	-
	MSEL-expressive language	1	0.76[b]	0.451	-
ASD	ADOS-Social Affect	2	0.19	0.661	-
	Vineland-Socialization	2	0.87	0.351	-
	Vineland-Motor Skills	2	0.03	0.869	-
	MSEL-Fine Motor	2	5.22	0.022*	0.02
	Vineland-Communication	2	0.13[b]	0.900	-
	MSEL-Receptive Language	2	1.39	0.239	-
	MSEL-expressive language	2	3.23	0.072	-
TD	ADOS-Social Affect	2	0.02	0.881	-
	Vineland-Socialization	2	0.20	0.658	-
	Vineland-Motor Skills	2	3.68	0.055	-
	MSEL-Fine Motor	2	0.77	0.380	-
	Vineland-Communication	2	0.09	0.769	-
	MSEL-Receptive Language	2	0.09	0.770	-
	MSEL-expressive language	2	0.34	0.558	-
ASD	ADOS-Social Affect	3	0.49	0.484	-
	Vineland-Socialization	3	0.92	0.337	-
	Vineland-Motor Skills	3	1.62	0.203	-
	MSEL-Fine Motor	3	2.50	0.114	-
	Vineland-Communication	3	0.10	0.747	-
	MSEL-Receptive Language	3	2.03	0.154	-
	MSEL-expressive language	3	2.50	0.114	-

Note. [a] = Statistic is Kruskal-Wallis chi-squared test and its reported effect size is Eta squared. [b] = Statistic is t-test and its reported effect size is Cohen's *d,* * = $P < .05$. All the tests are two-sided.

Supplementary Table 12 - Sex differences in SNF- external variables.

Group	*External Variable*	*Cluster*	*Statistic*[a]	*P-value*	*Effect size*
ASD	ADOS-Restricted and Repetitive Behavior	1	0.25	0.614	-
	CDI-WG-Words Produced	1	0.05	0.825	-
	CDI-WG-Words Understood	1	0.02	0.881	-
	CDI-WG-Early Gestures	1	-0.41[b]	0.686	-
	CDI-WG-Later Gestures	1	-1.54[b]	0.132	-
	CDI-WG-Total Gestures	1	-1.25[b]	0.220	
	CDI-WS-Words Produced	1	0.96	0.328	
	Geopref-Social % Fixation	1	0.00	0.950	-
TD	ADOS-Restricted and Repetitive Behavior	1	3.36	0.067	-
	CDI-WG-Words Produced	1	0.25	0.614	-
	CDI-WG-Words Understood	1	1.83	0.176	-
	CDI-WG-Early Gestures	1	4.89	0.027*	0.01
	CDI-WG-Later Gestures	1	2.57	0.109	-
	CDI-WG-Total Gestures	1	2.02[b]	0.044*	0.28
	CDI-WS-Words Produced	1	4.20	0.040*	0.01
	Geopref-Social % Fixation	1	0.75	0.386	-
ASD	ADOS-Restricted and Repetitive Behavior	2	0.02	0.878	-
	CDI-WG-Words Produced	2	1.99	0.158	-
	CDI-WG-Words Understood	2	2.10	0.147	-
	CDI-WG-Early Gestures	2	0.62[b]	0.537	-
	CDI-WG-Later Gestures	2	1.84	0.174	
	CDI-WG-Total Gestures	2	1.18	0.278	
	CDI-WS-Words Produced	2	1.17	0.279	-
	Geopref-Social % Fixation	2	0.01	0.925	-
TD	ADOS-Restricted and Repetitive Behavior	2	4.60	0.032*	0.22
	CDI-WG-Words Produced	2	-1.46[b]	0.258	-
	CDI-WG-Words Understood	2	-1.64[b]	0.195	-
	CDI-WG-Early Gestures	2	0.51	0.476	-
	CDI-WG-Later Gestures	2	0.35	0.741	-
	CDI-WG-Total Gestures	2	0.26	0.805	-
	CDI-WS-Words Produced	2	1.35	0.245	-
	Geopref-Social % Fixation	2	-0.02[b]	0.985	-
ASD	ADOS-Restricted and Repetitive Behavior	3	3.34	0.067	-
	CDI-WG-Words Produced	3	NEO	-	-
	CDI-WG-Words Understood	3	NEO	-	-
	CDI-WG-Early Gestures	3	NEO	-	-
	CDI-WG-Later Gestures	3	NEO	-	-
	CDI-WG-Total Gestures	3	NEO	-	-
	CDI-WS-Words Produced	3	7.96	0.005*	0.05
	Geopref-Social % Fixation	3	0.23[b]	0.824	-

Note. [a] = Statistic is Kruskal-Wallis chi-squared test and its reported effect size is Eta squared. [b] = Statistic is t-test and its reported effect size is Cohen's *d*, * = $P < .05$, NEO = Not enough observations for girls to examine sex differences. All the tests are two-sided.

Supplementary Table 13 - Sex differences across clusters.

Group	*Test*	X^2	*df*	*P-value*
ASD	Test of associations between sex and cluster membership	1.40	2	0.497
TD	Test of associations between sex and cluster membership	0.07	1	0.798

Note. The statistic is chi-squared test and all the tests are two-sided.

Supplementary Table 14 – Sex difference in income.

Group	*Statistic*[a]	*P-value*	*Effect size*
Overall	2.42	0.119	-
ASD	5.79	0.016*	< 0.01
TD	3.91	0.048*	0.01
DD	0.37	0.544	-

Note. [a] = Statistic is Kruskal-Wallis chi-squared test and its reported effect size is Eta squared, * = *P* < .05. All the tests are two-sided.

Supplementary Table 15 – SNF with sex as a main feature.

**ASD**				Social domain		Motor domain		Language domain				
Cluster	Diagnosis	Sex	Age	SA	SOC	MTR	FM	COM	RL	EL	N	
High (C2)	ASD	F	21.8	9.6	95.4	99.2	106.3	95	86.4	80.8	27	13.1%
	ASD	M	20.2	8.1	99	101.3	105.7	94.3	94.1	79.8	73	10.6%
Medium (C3)	ASD	F	25.4	14.9	84.9	91.7	82.3	76.3	48.9	53.6	143	69.4%
	ASD	M	25.9	13.7	82.9	92.1	81.2	75.4	54.8	54.7	512	74.6%
Low (C1)	ASD	F	27.5	17.2	68.3	78.4	61.3	56.4	24.3	35.2	36	17.5%
	ASD	M	29.6	17.8	67.2	80.5	57.3	52.7	21.6	29.9	101	14.7%
**TD**				Social domain		Moto domain		Language domain				
Cluster	Diagnosis	Sex	Age	SA	SOC	MTR	FM	COM	RL	EL	N	
High (C2)	TD	F	25.4	2.1	105.9	101.7	107.6	104.4	110.9	104.1	183	98.4%
	TD	M	27	2.3	103.4	100	102.1	102.7	105.2	102.1	250	96.5%
Medium (C3)	TD	F	23.1	6	98.3	88.3	87.3	84.7	83.2	67	3	1.6%
	TD	M	27.7	6.9	89.1	91.6	92.4	90.1	85.6	89.9	9	3.5%

Note. SA: ADOS Social Affect, SOC: Vineland Socialization, MTR: Vineland Motor Skills, FM: Std. MSEL Fine Motor, COM: Vineland Communication, RL: Std. MSEL Receptive Language, EL: Std. MSEL Expressive Language. Yellow highlighted cells show sex differences with darker color indicating higher means.

Supplementary Table 16 – Primary analysis means and SDs.

Test Subscale	*Group*	*Sex*	*Mean*	*SD*
CSBS Social	ASD	F	16.5	5.9
CSBS Social	ASD	M	14.7	5.9
CSBS Social	TD	F	19.6	5.2
CSBS Social	TD	M	18.5	5.4
CSBS Symbolic	ASD	F	9.4	3.5
CSBS Symbolic	ASD	M	8.1	3.5
CSBS Symbolic	TD	F	10.9	4.3
CSBS Symbolic	TD	M	10.3	3.9
CSBS Total	ASD	F	33.0	10.9
CSBS Total	ASD	M	28.9	10.9
CSBS Total	TD	F	39.1	11.7
CSBS Total	TD	M	37.0	11.2
CSBS Speech	ASD	F	7.1	2.8
CSBS Speech	ASD	M	6.3	3.0
CSBS Speech	TD	F	8.8	3.2
CSBS Speech	TD	M	8.2	3.2
WS Words Produced	ASD	F	55.4	90.9
WS Words Produced	ASD	M	58.4	97.2
WS Words Produced	TD	F	304.3	205.9
WS Words Produced	TD	M	244.1	186.5
WG Words Understood	ASD	F	46.5	48.7
WG Words Understood	ASD	M	34.6	44.4
WG Words Understood	TD	F	99.4	90.0
WG Words Understood	TD	M	80.5	75.1
WG Words Produced	ASD	F	4.9	8.8
WG Words Produced	ASD	M	2.9	8.0
WG Words Produced	TD	F	16.8	27.4
WG Words Produced	TD	M	11.9	19.8
WG Total Gestures	ASD	F	17.3	8.9
WG Total Gestures	ASD	M	16.2	8.9
WG Total Gestures	TD	F	29.8	13.4
WG Total Gestures	TD	M	25.8	11.9
WG Later Gestures	ASD	F	9.8	6.2
WG Later Gestures	ASD	M	9.1	6.3
WG Later Gestures	TD	F	18.4	10.4
WG Later Gestures	TD	M	15.3	8.8
WG Early Gestures	ASD	F	7.5	3.6
WG Early Gestures	ASD	M	7.0	3.5
WG Early Gestures	TD	F	11.4	3.9
WG Early Gestures	TD	M	10.5	3.8
Vineland Communication	ASD	F	75.5	15.6
Vineland Communication	ASD	M	74.5	15.8

Vineland Communication	TD	F	103.1	11.3
Vineland Communication	TD	M	101.1	9.9
Vineland Daily Living Skills	ASD	F	84.8	13.3
Vineland Daily Living Skills	ASD	M	82.8	14.0
Vineland Daily Living Skills	TD	F	103.7	11.4
Vineland Daily Living Skills	TD	M	99.8	11.4
Vineland Motor Skills	ASD	F	90.8	10.9
Vineland Motor Skills	ASD	M	91.1	10.9
Vineland Motor Skills	TD	F	100.1	9.6
Vineland Motor Skills	TD	M	100.0	10.4
Vineland Socialization	ASD	F	83.2	12.6
Vineland Socialization	ASD	M	82.0	12.6
Vineland Socialization	TD	F	104.9	9.5
Vineland Socialization	TD	M	102.9	10.8
Vineland ABC	ASD	F	80.2	11.6
Vineland ABC	ASD	M	79.1	12.2
Vineland ABC	TD	F	103.3	10.2
Vineland ABC	TD	M	101.0	9.8
MSEL Receptive Language	ASD	F	52.2	29.8
MSEL Receptive Language	ASD	M	54.8	30.0
MSEL Receptive Language	TD	F	108.2	19.3
MSEL Receptive Language	TD	M	103.4	18.1
MSEL Expressive Language	ASD	F	55.8	24.6
MSEL Expressive Language	ASD	M	54.1	24.6
MSEL Expressive Language	TD	F	101.4	18.8
MSEL Expressive Language	TD	M	99.0	15.3
MSEL Fine Motor	ASD	F	84.9	18.1
MSEL Fine Motor	ASD	M	83.0	18.1
MSEL Fine Motor	TD	F	107.8	14.9
MSEL Fine Motor	TD	M	105.0	23.9
MSEL Visual Reception	ASD	F	78.2	19.9
MSEL Visual Reception	ASD	M	80.2	21.4
MSEL Visual Reception	TD	F	113.6	18.6
MSEL Visual Reception	TD	M	110.0	17.4
ADOS SA	ASD	F	14.3	4.8
ADOS SA	ASD	M	13.8	4.7
ADOS SA	TD	F	2.4	2.0
ADOS SA	TD	M	2.9	2.5
ADOS R&R Behavior	ASD	F	4.7	2.2
ADOS R&R Behavior	ASD	M	4.9	2.1
ADOS R&R Behavior	TD	F	0.5	0.9
ADOS R&R Behavior	TD	M	0.6	0.9
ADOS Total	ASD	F	19.1	5.9
ADOS Total	ASD	M	18.7	5.7
ADOS Total	TD	F	2.9	2.3

ADOS Total	TD	M	3.5	2.9

Note. SD: Standard Deviation.

Supplementary Table 17 - Primary analysis of sex differences in ASD and TD toddlers across ADOS modules.

Module	*Subscale*	*Mean Diff*	*Statistic*[a]	*P-value*	*Adj P-value*	*Effect size*
Toddler (N=1228)	ASD-Social Affect	0.91	8.33	0.004	0.024*	<0.01
	TD-Social Affect	-0.6	4.65	0.031	0.093	-
	ASD-Restricted & Repetitive Behavior	0	0.01	0.943	0.985	-
	TD-Restricted & Repetitive Behavior	-0.19	4.5	0.034	0.204	-
	ASD-Overall Total	0.91	5.22	0.022	0.066	-
	TD-Overall Total	-0.78	6.52	0.011	0.066	-
Module 1 (N=372)	ASD-Social Affect	-0.41	0.62	0.43	0.860	-
	TD-Social Affect	-0.05	0.02	0.9	0.900	-
	ASD-Restricted & Repetitive Behavior	-0.39	2.32	0.128	0.268	-
	TD-Restricted & Repetitive Behavior	0.18	0	0.985	0.985	-
	ASD-Overall Total	-0.8	1.93	0.165	0.330	-
	TD-Overall Total	0.14	0	0.978	0.978	-
						-
Module 2 (N=205)	ASD-Social Affect	0.26	0.2[b]	0.84	0.900	-
	TD-Social Affect	-0.17	0.14	0.704	0.900	-
	ASD-Restricted & Repetitive Behavior	-0.98	2.24	0.134	0.268	-
	TD-Restricted & Repetitive Behavior	0.02	0.06	0.802	0.985	-
	ASD-Overall Total	-0.71	-0.51[b]	0.612	0.898	-
	TD-Overall Total	-0.15	0.1	0.748	0.898	-

Note. [a] = Statistic is Kruskal-Wallis chi-squared test and its reported effect size is Eta squared. [b] = Statistic is t-test and its reported effect size is Cohen's *d*, * = $P < .05$. Mean diff = mean (f) – mean (m). Adj P-value = Multiple tests were corrected by FDR. All the tests are two-sided.

Supplementary Table 18 - Primary analysis of sex differences in DD toddlers across ADOS modules.

Module	*Subscale*	*Mean Diff*	*Statistic*[a]	*P-value*	*Adj P-value*	*Effect size*
Toddler (N=259)	Social Affect	1.37	4.95	0.026	0.078	-
	Restricted & Repetitive Behavior	-0.24	2.58	0.109	0.164	-
	Overall Total	1.13	1.88	0.171	0.513	-
Module 1 (N=174)	Social Affect	0.52	0.09	0.764	0.764	-
	Restricted & Repetitive Behavior	-0.57	4.58	0.032	0.096	-
	Overall Total	-0.07	0.28	0.596	0.761	-
Module 2 (N=42)	Social Affect	0.3	0.25	0.615	0.764	-
	Restricted & Repetitive Behavior	-0.08	0.00	0.979	0.979	-
	Overall Total	0.21	0.09	0.761	0.761	-

Note. [a] = Statistic is Kruskal-Wallis chi-squared test and its reported effect size is Eta squared. [b] = Statistic is t-test and its reported effect size is Cohen's *d*, * = $P < .05$. Mean diff = mean (f) – mean (m). Adj P-value = Multiple tests were corrected by FDR. All the tests are two-sided.

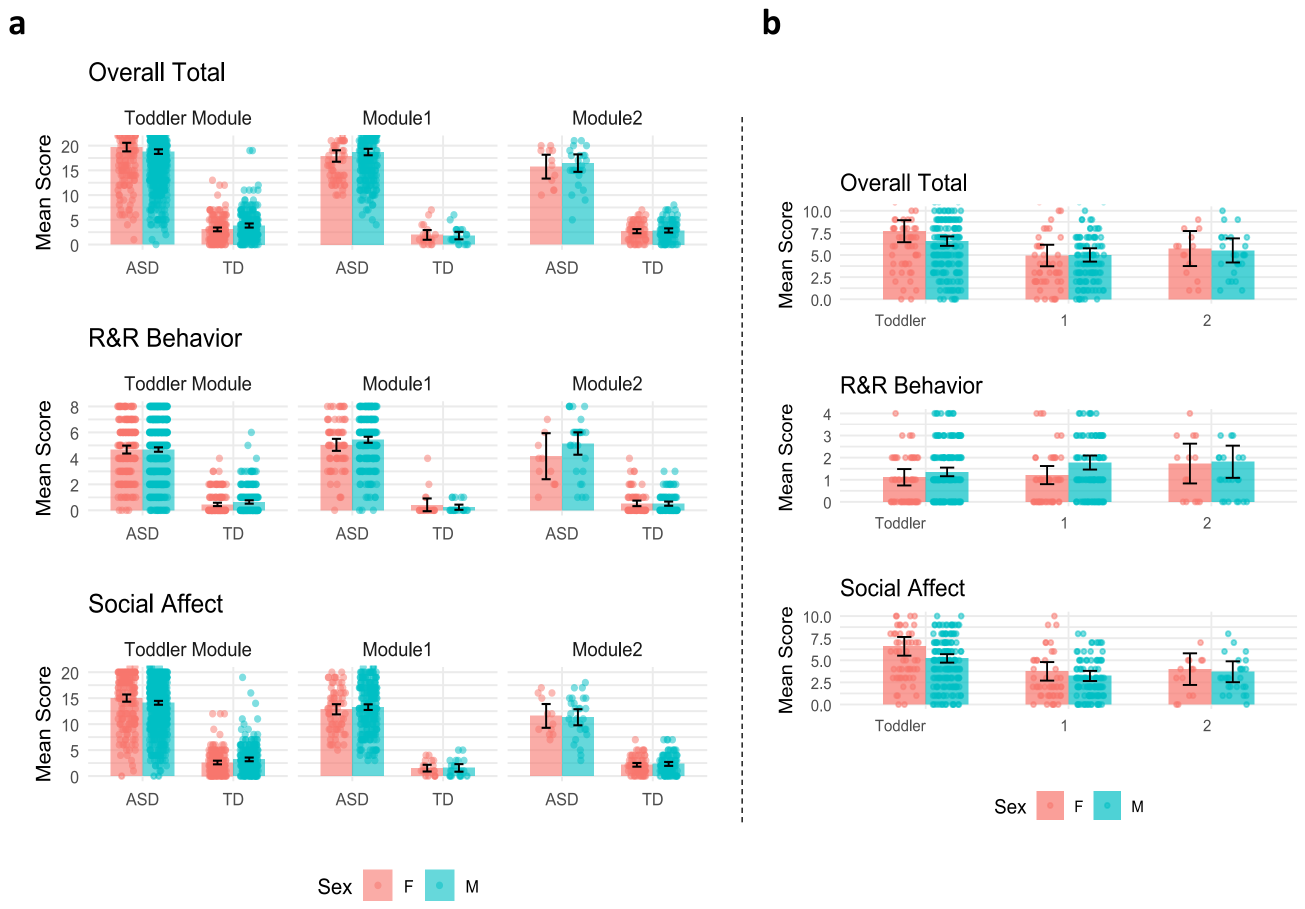

Supplementary Figure 1. **Comparison of sex differences in ASD vs TD, and DD groups**. Panel **a** illustrates mean scores for the ASD and TD groups across modules (Toddler Module, Module 1, Module 2) in three subscales: Overall Total, R&R Behavior, and Social Affect. Panel **b** depicts the corresponding results for the DD group. Data are presented as mean values ± confidence intervals. Refer to Supplementary Tables 17 and 18 for sample sizes.
