# Supplementary material for: Large-scale examination of early-age sex differences in neurotypical toddlers and those with autism spectrum disorder or other developmental conditions
Source: Nat Hum Behav. 2025 May 26;9(8):1697–709. doi: 10.1038/s41562-025-02132-6 (PMC12367553; doi:10.1038/s41562-025-02132-6)
Supplement: Supplementary file 2 — Reporting Summary [file 41562_2025_2132_MOESM2_ESM.pdf]

nature portfolio

Corresponding author(s): Karen Pierce
Last updated by author(s): Jan 6, 2025

Reporting Summary

Nature Portfolio wishes to improve the reproducibility of the work that we publish. This form provides structure for consistency and transparency in reporting. For further information on Nature Portfolio policies, see our Editorial Policies and the Editorial Policy Checklist.

Statistics

For all statistical analyses, confirm that the following items are present in the figure legend, table legend, main text, or Methods section.

n/a	Confirmed	
☒	☐	The exact sample size (*n*) for each experimental group/condition, given as a discrete number and unit of measurement
☒	☐	A statement on whether measurements were taken from distinct samples or whether the same sample was measured repeatedly
☐	☒	The statistical test(s) used AND whether they are one- or two-sided<br>*Only common tests should be described solely by name; describe more complex techniques in the Methods section.*
☐	☒	A description of all covariates tested
☐	☒	A description of any assumptions or corrections, such as tests of normality and adjustment for multiple comparisons
☐	☒	A full description of the statistical parameters including central tendency (e.g. means) or other basic estimates (e.g. regression coefficient) AND variation (e.g. standard deviation) or associated estimates of uncertainty (e.g. confidence intervals)
☐	☒	For null hypothesis testing, the test statistic (e.g. *F*, *t*, *r*) with confidence intervals, effect sizes, degrees of freedom and *P* value noted<br>*Give P values as exact values whenever suitable.*
☒	☐	For Bayesian analysis, information on the choice of priors and Markov chain Monte Carlo settings
☐	☒	For hierarchical and complex designs, identification of the appropriate level for tests and full reporting of outcomes
☐	☒	Estimates of effect sizes (e.g. Cohen's *d*, Pearson's *r*), indicating how they were calculated

Our web collection on statistics for biologists contains articles on many of the points above.

Software and code

Policy information about availability of computer code

Data collection	The study data collection was based on 1-Year Well Baby Check Up Approach (Pierce et al., 2011) and Get SET Early model (Pierce et al., 2021).
Data analysis	All the data cleaning and the majority of analyses were conducted in R and Longitudinal analyses were conducted using Mplus version 8.3.

For manuscripts utilizing custom algorithms or software that are central to the research but not yet described in published literature, software must be made available to editors and reviewers. We strongly encourage code deposition in a community repository (e.g. GitHub). See the Nature Portfolio guidelines for submitting code & software for further information.

Data

Policy information about availability of data

All manuscripts must include a data availability statement. This statement should provide the following information, where applicable:
- Accession codes, unique identifiers, or web links for publicly available datasets
- A description of any restrictions on data availability
- For clinical datasets or third party data, please ensure that the statement adheres to our policy

The data supporting the findings of this study are available on the ACE lab's GitHub page. Link: https://github.com/ACE-UCSD/Autism-Sex-Differences-Analysis-Pathway.

Research involving human participants, their data, or biological material

Policy information about studies with human participants or human data. See also policy information about sex, gender (identity/presentation), and sexual orientation and race, ethnicity and racism.

Reporting on sex and gender	The findings of this study are applicable solely to sex, as investigating the sex differences in toddlers with autism was the primary objective. Our data stratify all subjects by sex, which is indicated in a separate column on the primary datasheet. It should be noted that, because the study involves toddlers, sex was determined based on parental reports. Consent has been obtained to share individual-level data that has been de-identified.
Reporting on race, ethnicity, or other socially relevant groupings	All of the data collected in the current study was funded by national and state health agencies, and the categorizations of race and ethnicity are based on NIH guidelines.
Population characteristics	Please see Table 1 in the manuscript for detailed information regarding population characteristics.
Recruitment	Approximately 75% of the sample was collected using a general population-based screening approach called Get Set Early which minimizes recruitment biases because all toddlers are screened with parent report questions at pediatric well-baby check ups at 12:18 and 24 months.
Ethics oversight	This research has met all the ethical requirements regarding human research protection program under the approval of the UCSD Office of IRB administration (project number: 202115).

Note that full information on the approval of the study protocol must also be provided in the manuscript.

Field-specific reporting

Please select the one below that is the best fit for your research. If you are not sure, read the appropriate sections before making your selection.

☐ Life sciences ☒ Behavioural & social sciences ☐ Ecological, evolutionary & environmental sciences

For a reference copy of the document with all sections, see nature.com/documents/nr-reporting-summary-flat.pdf

Behavioural & social sciences study design

All studies must disclose on these points even when the disclosure is negative.

Study description	The study is quantitative and includes cross-sectional, cluster, and longitudinal analyses.
Research sample	A total of 2,618 toddlers participated and included those with a diagnosis of ASD (N=1539; 1200 M and 339 F; mean age 28.6 months), DD (N=478; 349 M and 129 F; mean age 26.0 months) as well as those who were TD (N=601; 349 M and 252 F; mean age 25.7 months).
Sampling strategy	The study data collection was based on 1-Year Well Baby Check Up Approach (Pierce et al., 2011) and Get SET Early model (Pierce et al., 2021).
Data collection	The study data collection was based on 1-Year Well Baby Check Up Approach (Pierce et al., 2011) and Get SET Early model (Pierce et al., 2021).
Timing	2002-2022
Data exclusions	Data were excluded based on criteria such as deafness, blindness, or less than 50% exposure to English or Spanish.
Non-participation	Data from families who declined to participate or dropped out of the study were not included in the analysis. Since this study encompasses a combination of data collected over an 11-year period, tracking can only be reported approximately. We estimate that less than one percent of the participating families dropped out of the study.
Randomization	Participants were assigned to groups based on their sex and diagnosis, in alignment with the study's objectives.

Reporting for specific materials, systems and methods

We require information from authors about some types of materials, experimental systems and methods used in many studies. Here, indicate whether each material, system or method listed is relevant to your study. If you are not sure if a list item applies to your research, read the appropriate section before selecting a response.

Materials & experimental systems

n/a	Involved in the study
☒	☐ Antibodies
☒	☐ Eukaryotic cell lines
☒	☐ Palaeontology and archaeology
☒	☐ Animals and other organisms
☒	☐ Clinical data
☒	☐ Dual use research of concern
☒	☐ Plants

Methods

n/a	Involved in the study
☒	☐ ChIP-seq
☒	☐ Flow cytometry
☒	☐ MRI-based neuroimaging

Plants

Seed stocks	*Report on the source of all seed stocks or other plant material used. If applicable, state the seed stock centre and catalogue number. If plant specimens were collected from the field, describe the collection location, date and sampling procedures.*
Novel plant genotypes	*Describe the methods by which all novel plant genotypes were produced. This includes those generated by transgenic approaches, gene editing, chemical/radiation-based mutagenesis and hybridization. For transgenic lines, describe the transformation method, the number of independent lines analyzed and the generation upon which experiments were performed. For gene-edited lines, describe the editor used, the endogenous sequence targeted for editing, the targeting guide RNA sequence (if applicable) and how the editor was applied.*
Authentication	*Describe any authentication procedures for each seed stock used or novel genotype generated. Describe any experiments used to assess the effect of a mutation and, where applicable, how potential secondary effects (e.g. second site T-DNA insertions, mosiacism, off-target gene editing) were examined.*
